# Social cognitive career theory: The experiences of Korean college student-athletes on dropping out of male team sports and creating pathways to empowerment

**DOI:** 10.3389/fpsyg.2022.937188

**Published:** 2022-09-27

**Authors:** Benjamin H. Nam, Racheal C. Marshall

**Affiliations:** ^1^School of Education, Shanghai International Studies University, Shanghai, China; ^2^Graduate and Professional Studies in Education, California State University, Sacramento, Sacramento, CA, United States

**Keywords:** college sport, career transition out of the sport, counselor education, critical approach, phenomenologhy

## Abstract

The South Korean elite sport system is facing a wide range of problems that account for the high dropout rate among college student-athletes. However, research on dropout rates of student-athletes is so far been limited, which amplifies the actual voices of this group, their dropout experiences, and their challenges, while they were in the career transition process. Therefore, this study used a critical phenomenological approach as a primary methodological lens to gather information on 15 formal Korean male college student-athletes on dropping out of team sports, exploring their life challenges during their career transitions out of the sport. The result showed two main thematic categories with sub-themes, which include (1) factors affecting burning out and terminating athletic careers: (a) injury and failure of rehabilitation and (b) bullying and abandonment; and (2) factors hindering post-retirement career advancement: (a) prejudice and exclusion and (b) absence of mentors and counselors. This study used Social Cognitive Career Theory to explore the participants' progression through specific interventions that engage and empower. Overall, the current study calls upon researchers, counselors, and administrators to continue exploring advocacy efforts with this population to alter policy and practice.

## Introduction

A growing body of literature has explored elite athletes' life course stages to understand the interaction of sport dropout and burnout factors with issues involving athletes' transition out of the sport (Park et al., 2013; Crane and Temple, 2015; Eime et al., 2019). According to Stambulova and Alfermann (2009), elite athletes' careers generally entail six prominent stages: “(1) the beginning of sport specialization, (2) the transition to more intensive training in a chosen sport, (3) the transition from junior to the senior sport, (4) the transition from amateur to professional sport, (5) the transition from the peak to the final stage of the athletic career, and (6) the transition to the post-career” (p. 296). Considering the competitive nature of the elite sport, numerous adolescent and young college student-athletes drop out of athletic programs (henceforth: sport dropouts) before reaching top performances or accomplishing top athletic careers, as a relatively fewer number can continue competing at national, international, and professional levels (Fraser-Thomas et al., 2005, 2008; Cervelló et al., 2007; Wall and Côté, 2007; Calvo et al., 2010).

Sport dropouts are vulnerable, experiencing a series of life challenges, such as obesity, self-isolation, and drug and alcohol abuse, without social and emotional support (Crane and Temple, 2015). Further, they are anxious about career advancement after retiring from sports due to “feelings of failure or self-disappointment,” and they have difficulties building “positive self-image and self-confidence” (Park et al., 2013, p. 44). The most serious factors influencing athletes' career transition are financial issues, identity crisis, and social exclusion. These issues ultimately prevent them from staying mentally and physically healthy, which can potentially lead to suicidal ideation (Cosh et al., 2013; Roberts et al., 2015).

Scholars have proposed influential theoretical frameworks or conceptual models, such as the self-determination theory (e.g., Calvo et al., 2010), achievement goal theory (e.g., Cervelló et al., 2007), leisure constraints theory (e.g., Crane and Temple, 2015), and various theories of motivation and its applied concepts (e.g., Fraser-Thomas et al., 2005, 2008), to explore the sport dropouts' life course stages. Among the many theories and conceptual maps, counselors can use Social Cognitive Career Theory (SCCT) to examine life transitions and career development (Lent et al., 1994). SCCT outlines both internal motivations and external barriers and their effects on individuals' career development. SCCT attempts to outline the relationships between values, needs, aptitudes, and interests and their effects on career development. SCCT then outlines how these internal motivations are immersed in social processes (Lent et al., 1994, 2000). Although SCCT is a prominent conceptual map to explore challenges and opportunities for individuals' career prospects, scholars have paid scant attention to using this theoretical framework to interpret factors that impact burnout and termination of athletic careers and hinder the effectiveness of career transitions out of the sport, especially analyzing the empirical voices of those college sport dropouts who have limited experiences with career mentoring and counseling programs.

The case of South Korea and its national sport governance—the Korea University Sport Federation (KUSF)—provides a prominent instance, which overlooks career concerns of their college sport dropouts, especially those who participated in male team sports including soccer, baseball, basketball, volleyball, and ice hockey, as they are no longer affiliated with their athletic programs (KUSF, 2017, 2018, 2019). Thus, the current study argues that these populations can possibly face numerous forms of life challenges in their career transition experiences without sufficient social and emotional support. Given the context, SCCT can be useful to identify both challenges and opportunities for their career prospects and provide practical implications for their national college sport authority (i.e., KUSF) and their higher education institutions (i.e., member universities). Therefore, using SCCT and a critical phenomenological approach, this study examined the life course stages of 15 Korean male college sport dropouts at a time when they end their athletic careers before exhausting their eligibility and during their career transitions out of the sport. Overall, this paper aims to gain a more in-depth theoretical understanding, provide practical implications, and expand the knowledge into a global scholarship from East Asian perspectives.

### Social cognitive career theory and practice

**Figure 1:**
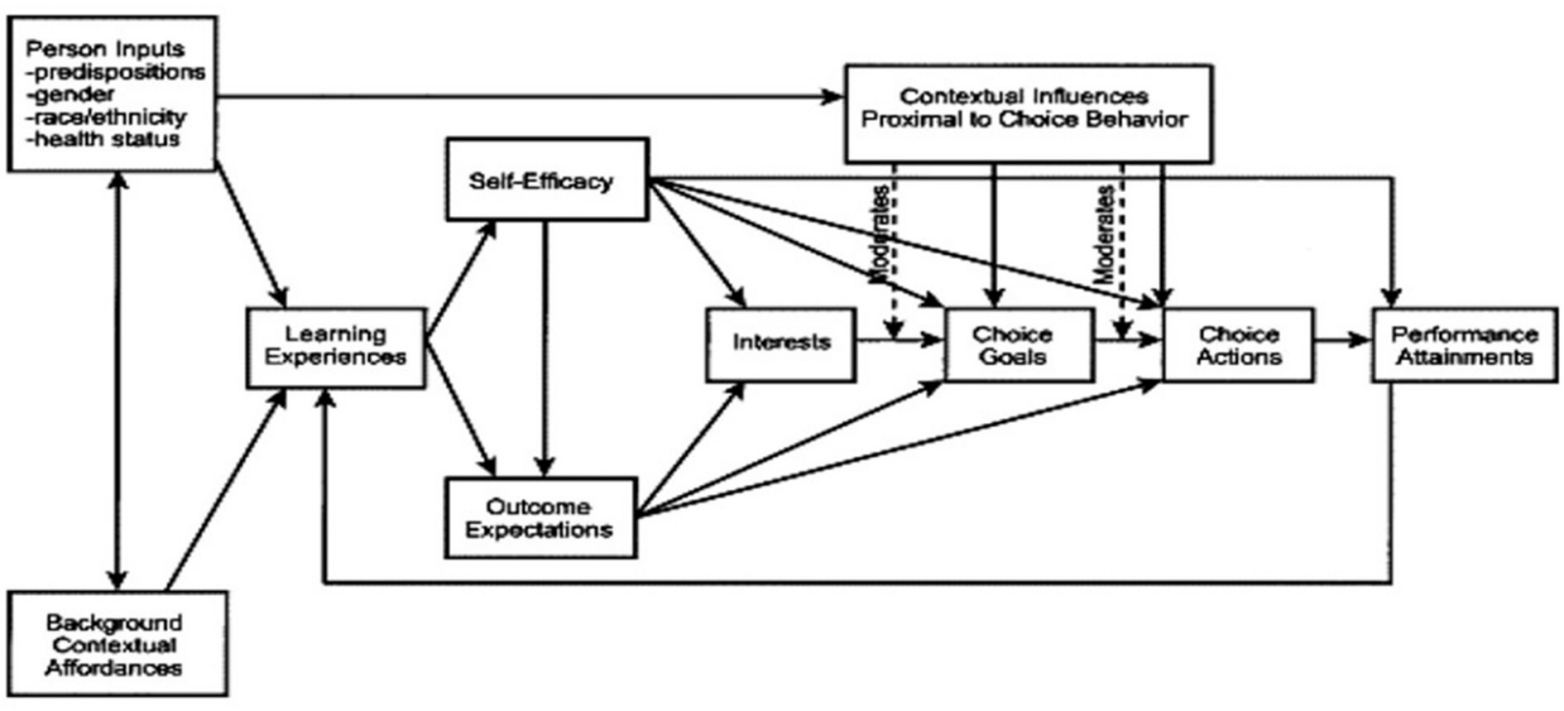
Social cognitive career theory.

Social Cognitive Career Theory (SCCT) has foundations in Albert Bandura's (1986) cognitive theory explaining triadic reciprocity as a bidirectional model of causality. SCCT uses the bidirectional dynamic to outline key theoretical constructs: (a) personal (motivations, health, predispositions), (b) behavioral (decision and experiences), and (c) environmental (social and cultural surroundings; Hackett and Lent, 1992). Human functioning must be considered and explored within a context, as a person's cognitions, genetic predispositions, and specific environment interact to produce career interests, goals, and decisions (Wang et al., 2007). In this reciprocal relationship, learning is influenced by and influences self-efficacy beliefs, which affect outcome expectations and career decisions (Figure 1). Therefore, people are both “products and producers of their environment” in this reciprocal process (Bandura and Wood, 1989, p. 362).

As outlined in Figure 1, personal inputs, context, and learning experiences create a reciprocal triangle and then expand outward to influence self-efficacy and outcome expectations. Self-efficacy is the main construct of SCCT. It is defined as “people's judgments of their capabilities to organize and execute courses of action required to attain designated types of performances” (Bandura, 1986, p. 391). Higher levels of self-efficacy are shown to affect behavioral choices (Saks and Ashforth, 2000), SCCT suggests that efficacy expectations result in specific outcomes needed to overcome barriers to obtaining vocational interests, goals, and behaviors (Lent et al., 1994, 2000). Self-efficacy and outcome expectations then affect career interests, goals, actions, and performance with contextual variables moderating these choices. Therefore, regardless of a career decision, the context must be considered.

Cunningham et al. (2005) explored the relationship between SCCT variables and career choices of 197 students engaged in sport and leisure activities. They found that for this specific population, self-efficacy was related to both outcome expectation and satisfaction with vocational interests and that contextual influences affected self-efficacy. These results supported the theory outlining those contextual barriers and support to career entry are internalized and affect career decisions. Due to SCCT's focus on the context, including cultural, political, economic, social, proximal (e.g., family and school), and geographic, it is well suited to provide an outline for investigation and intervention. The effect of the environment is important as this study explores the experiences of college sport dropouts in the social and cultural context of sport in Korea.

Taken in the context of this research, college sport dropouts have contextual and personal factors that decrease their self-efficacy and change their outcome expectations for sport continuation. With the lack of support, their self-efficacy suffers, and they no longer view sport as a viable career option altering their interests, goals, and actions. Multiple contextual factors can influence self-efficacy, including seeing similar individuals succeeding in a specific role (Quimby and Desantis, 2006; Moran-Miller and Flores, 2011), their working hours of these students (Moran-Miller and Flores, 2011), paternal support and access of information (Flores and O'Brien, 2002), and academic choices (Lent et al., 2008). Given the historical context, this study can provide implications for wellbeing and burnout during the career development process. Furthermore, Sheu et al. (2017) explored the wellbeing of 757 college students in China. The results highlighted that outreach programs should focus on gathering support, increasing self-efficacy, and facilitating academic goal setting to decrease burnout. College sport dropouts face similar academic and social contextual barriers that affect their self-efficacy and career decisions that lead to dropping out, understanding their experiences can help to develop specific interventions to aid in career development.

### The rationale for applying SCCT in Korean college sports dropouts' life course stages

The person-environment interaction is significant to this study, as elite athletes' career development is inescapably connected to both the internal cognitive processes linked to self-efficacy and the surrounding social environment (Lent and Brown, 2013). As elite athletes' transition through junior, amateur, professional, peak, and post-career stages, a dynamic interplay between social support, cultural influences, and self-efficacy affects their decisions, such as dropping out. Hence, SCCT creates a framework to better outline the decision-making processes of elite athletes and with it how to better support them in their contextual background of Korea.

Additionally, SCCT provides a guide to understand the conditions that motivate self-directed career behaviors, such as training, networking, and studying (Hirschi, 2018). Career behaviors gathered from direct or vicarious learning experiences promote satisfaction and wellbeing at work (Lent and Brown, 2013). By understanding the conditions that affect career behaviors, such as dropout, counselors, teachers, coaches, and social support, can use SCCT to guide and structure interventions that are specific to the clients' experiences and context (Lent and Brown, 2013). For college sport dropouts, SCCT can be used to outline their career development process and provide opportunities for intervention and support. Based on the conceptual maps of SCCT, the following research questions guided this study are:

RQ1: What factors contributed to burnout and termination of Korean college sport dropouts' athletic careers?

RQ2: What factors hindered the effectiveness of Korean college sport dropouts' career transitions out of sport?

## Method

### Research context

This study explores the life experiences of 15 Korean male college sport dropouts who were affiliated with the KUSF member institutions. Briefly informing regarding the contextual background of the study, individual sports (e.g., winter sports, wrestling, judo, swimming, and archery) and female sports are governed by the Korean Sport and Olympic Committee (KSOC) or national sports governing bodies of each sport. The KUSF was established by the Ministry of Culture, Sport, and Tourism in 2010. To date, the national college sport governance has been overseeing a total of 104 member universities, primarily organizing male team sports including soccer, baseball, basketball, volleyball, and ice hockey. The KUSF has been making endeavors to boost the commercialization of intercollegiate athletics through male team sports such as media rights, ticket sales, promotion, and donations from alumni or local enterprises (KUSF, 2017, 2018, 2019).

Concerning college sport dropouts, an archival record issued by the KUSF stated that as of 2016, about 46,000 youth athletes across ~2,300 athletic programs were seeking college admissions with athletic scholarships. However, only 6,780 college student-athletes were affiliated with 98 KUSF member universities (KUSF, 2017). According to another archival record provided by KUSF (2018), 45% dropped out of athletic programs before their junior year. Additionally, government research reports conducted by the Korean Sport and Olympic Committee (KSOC) and Presidential Committee on Young Generation (PCYG) showed that there were 388,412 former elite student-athletes between 2004 and 2016 ranging in age from their 20–40 s. Of the ~13,000 former athletes who participated in the survey, more than 65% indicated that they are unemployed or hold a non-permanent employment position, and they represented themselves as working class. The result showed that 56.3% terminated their sports spontaneously, while the remaining 43.7% terminated their sports reluctantly (e.g., pressure from coaches, peers, and parents). The most common and influential burnout factors were uncertain future career, injury, limited athletic skills, and abusive culture (PCYG, 2015; KSOC, 2017).

Despite recent archival data and governmental research reports and documentation on dropout rates of Korean college student-athletes indicated some common dropout and burnout factors, highlighting limitations to obtaining white-collar and full-time employment opportunities, little research has been conducted on the actual voices of this underrepresented group of athletes and their experiences in their life course stages. Therefore, this study examined the life course stages of 15 Korean college sport dropouts, identifying factors influencing burnout and hindering the effectiveness of their career transition out of the sport.

### A critical phenomenological approach

This qualitative study adopted a critical phenomenological approach from philosophical and methodological perspectives (van Manen, 1990; Moustakas, 1994; Creswell, 2013; Merriam and Tisdell, 2016). Accordingly, this approach is a prominent qualitative paradigm to manage the information collected from marginalized groups, such as social categorization data pertaining to social class, race, ethnicity, gender, and sexual orientation. Hence, investigators contemplated how their scholarship can advocate for socially marginalized individuals, motivating them to raise their critical voices regarding unfairness and inequity (Creswell, 2013). Moreover, a phenomenological approach can be used as an analytical and interpretive lens to view life challenges of study subjects who encounter emotional unrest, trauma, anxiety, and anger and identify factors that influence these situations from both socio-cultural and socio-psychological perspectives (Moustakas, 1994). Thus, the investigators interpret their information, such as interview transcripts, by deducing participants' feelings about and perceptions of a particular social phenomenon (van Manen, 1990). Pertinent to the current study, the authors perceived that Korean college sport dropouts are vulnerable and socially marginalized from both athletic society and general society in Korea, facing diverse life challenges and emotional unrest during their career transition process (PCYG, 2015; KSOC, 2017).

From socio-cultural perspectives, those successful athletes such as Olympians, international and national champions, and professional athletes have more opportunities to obtain financial support from the government and participate in career assistance programs such as language training, life skill training, and vocational education and training programs both in public and private sectors. However, college sports dropouts can potentially be excluded to gain those benefits. They usually terminate their athletic careers without specific future career plans. Even though they are willing to develop new academic habits and life skills, it can be challenging to accomplish their career goals without specific career mentoring and counseling programs, especially financial factors that can help obtain their desired career paths (Park et al., 2012; Lim et al., 2015; Kim et al., 2020; Kim and Dawson, 2021; Kim and Tak, 2021). These populations can face diverse emotional challenges, while they are struggling to overcome diverse life barriers. Therefore, a critical phenomenological approach can serve as a useful methodological lens in this study.

### Participants

Following the Institutional Research Board (IRB) approval from the primary investigator's institution, the authors used purposive and snowball sampling methods to recruit 15 former Korean male college student-athletes who dropped out of athletic programs to conduct semi-structured interviews^1^. The interviews took place in Korea between May 2018 and July 2018. The participants formerly played five different team sports, including baseball (*n* = 2), basketball (*n* = 3), soccer (*n* = 7), volleyball (*n* = 2), and ice hockey (*n* = 1; see Table 1). The participants were in their 30–40 s. They held full-time positions in diverse professional fields. However, every participant faced life difficulties and vocational challenges in their entire 20 s through early 30 s. Namely, they testified that they often felt socially marginalized and prejudiced, being perceived as uneducated or ignorant during the career transition process. Interview times ranged from 25 to 65 min and took place in public, such as coffee shops or lounges, near the participants' workplaces or residential areas.

**Table 1:** Demographic information of the participants.

**Pseudonym**	**Age**	**Gender**	**Sport**	**Length of athletic career**	**Dropout period**
An-Min	32	Male	Soccer	13 Years	Senior
Bang-Jin	36	Male	Soccer	7 Years	Freshman
Byung-Man	34	Male	Basketball	14 years	Senior
Chul-Sang	34	Male	Soccer	12 years	Senior
Dae-Chan	38	Male	Soccer	15 Years	Junior
Dong-Woo	30	Male	Soccer	14 years	Junior
Eun-Woo	41	Male	Baseball	7 years	Freshman
Guen-Joo	39	Male	Basketball	11 years	Senior
Hyo-Chan	34	Male	Soccer	9 years	Freshman
In-Sung	31	Male	Soccer	13 years	Senior
Jun-Jae	38	Male	Ice Hockey	10 years	Sophomore
Min-Jong	38	Male	Baseball	10 years	Sophomore
Sang-Jun	35	Male	Volleyball	9 years	Freshman
Tae-Min	34	Male	Volleyball	9 years	Junior
Woo-Sung	35	Male	Basketball	7 years	Freshman

### Data analysis

The authors read all transcripts and coded the interview responses to deduce the most common and useful themes that would be exhaustive, mutually exclusive, sensitive, and conceptually represented the interviewers' responses to the proposed questions (Merriam and Tisdell, 2016). A thematic analysis method was conducted following Strauss and Corbin's approach, which is useful to analyze particular social phenomena in a systemic way that deduces conclusive results in credible responses to the research questions (Strauss and Corbin, 1990). Notably, in phenomenology, Zahavi (2019) stated that “thematic change is exactly based on the fact” that “theme is situated in a field that is co-given” in which phenomenologists consider exploring the subsequent thematic experiences of individuals as an object (p. 11). In this context, the authors in the current study “grasp the emotions or experiences” of the participants, attempting to establish main thematic categories with sub-themes through semi-structured interview transcripts (Zahavi, 2019, p. 82). Accordingly, the main questions and probing questions in the interview protocol entail their life course stages ever since they initially participated in their specialized sport programs (see Table 2).

**Table 2:** Interview protocol.

1. Describe what the Athletic Specialist System in South Korea is.
a. How long did you play your sport?
b. What was your initial motivation to engage in your sport?
c. Tell me a story about your experiences as an athletic specialist in South Korea?
d. What were your goals as an athletic specialist?
e. Tell me the athletic culture you experienced.
2. Describe what impacted you to terminate your athletic career and what challenges did you face.
a. What specifically made you consider dropping out of your athletic programs?
b. Tell me a story about your experiences as a dropout college student-athletes?
3. Describe if you had any experiences regarding unequal treatment or felt marginalized as a dropout college student-athletes?
a. Tell me a story about your social networking experiences after you terminated your athletic career.
b. Tell me a story about your academic engagement after you terminated your athletic career.
4. Tell me how you overcame diverse social and cultural barriers
a. Tell me a story about your life experiences after you graduated from college.
b. Tell me a story about your social networking experiences after your graduation.
c. Tell me a story about your educational experiences after graduation.
5. Describe how you prepared for your current employment?
a. Tell me a story about your experiences to gain new knowledge or skills through career assistance programs if you had experiences.
b. What were the most important knowledge or skills to obtain your current employment?
c. In what ways did you specifically gain and develop new knowledge and skills?
d. Who financially supported you to develop new knowledge and skills?
e. Who emotionally supported you to develop new knowledge and skills?

In the initial stage, the authors reviewed the transcripts, focusing on the overall life stories. In this initial process, the authors identified several prominent life timeframes: (a) the youth period (8–12) characterized by talent identification, motivation to engage in sports, and athletic goal setting; (b) the adolescent period (13-18) characterized by skill development and priority of athletics over academics; and (c) the young adult and post-retirement period (19+) characterized by dropout factors, identity crisis, life challenges without emotional and social support, social prejudice, exclusion, stigmatization, and traumatization during schooling and career transition out of the sport. Furthermore, the authors reviewed the transcripts and focused more on coding specific issues and events alongside the participants' life challenges and emotional distress. Notably, participants commonly expressed that their career transition process was difficult without emotional and social support.

Additionally, the authors reviewed the transcripts multiple times again and considered more common and useful themes aligned with the concepts of the SCCT. Accordingly, two primary themes and their entailing sub-themes emerged: (1) factors influencing burning out and terminating athletic careers, specifically (a) injury and failure of rehabilitation and (b) bullying and abandonment, and (2) factors hindering the effectiveness of career transitions out of the sport, particularly (a) prejudice and exclusion and (b) absence of mentors and counselors. The findings and analysis are presented through a descriptive and interpretive analysis technique that represents the actual voices of the participants, along with the chosen theoretical framework (Merriam and Tisdell, 2016). Overall, considering the research purpose and its questions, this study focused solely on analyzing the life challenges of the participants during the period when they end their athletic careers before exhausting their eligibility and during post-retirement career advancement.

## Results

This study examined the experiences of Korean male college sport dropouts and determined if there were any factors that influence life challenges that hampered them from accomplishing their career goals. In the overall coding procedure, the authors drew ~250 quotes and verbatims from the interview information and represented two main thematic categories with two sub-themes each, which include (1) factors affecting burning out and terminating athletic careers: (a) injury and failure of rehabilitation and (b) bullying and abandonment; and (2) factors hindering post-retirement career advancement: (a) prejudice and exclusion and (b) absence of mentors and counselors (see Table 3).

**Table 3:** Summary of main and sub-themes, illustrative core ideas, and examples of code.

**Main thematic categories/sub-themes**	**Illustrative core ideas/examples of code**	**Frequency**
1. Factors affecting burning out and terminating athletic careers		
a. Injury and failure of rehabilitation	Most participants shared that they had chronic injuries, whether these were severe or minor. These factors significantly decreased their motivations to sustain their athletic careers.	General
	Example: I injured my back because one of my seniors hit me, so I couldn't continue my athletic career. My back is better now, but at that time, it was a serious injury…It was unfortunate that I injured my back. I had to take enough rest and recuperate, but my seniors told me not to talk about the issue to my coach. And, my coach kept having me participate in practice. So, I burnt out and quit baseball. (Min-Jong)	
b. Bullying and Abandonment	More than half of the participants witnessed or experienced bullying and abandonment, which contributed to burning out and terminating their athletic careers.	Typical
	Example: If juniors are against seniors; they can be isolated. That was common. Coaches usually take the seniors' sides. It's like this. If you cannot endure the abusive culture, you need to quit your sport. (Chul-Sang)	
2. Factors hindering post-retirement career advancement		
a. Prejudice and exclusion	All participants directly experienced certain forms of prejudice and exclusion in schooling after they left their sports teams. These issues continued after college graduation. Some of the participants shared negative experiences of job interviews or graduate school application interviews.	General
	Example: When student-athletes quit their sports, they attempted to come to class more often and tried to do their best to adjust to the new academic culture and environment. However, they often became discouraged because people outside of the athletic society frequently view that athletes cannot do well academically and socially without any logical reasons…This is typical that the degree of social prejudice or stereotype about [dropout] student-athletes is even more excessive when we apply for jobs. They are certainly vulnerable and easily screened during the job application process. For these reasons, many of them give up on their competitive occupational careers, being remained losers in society. (Jun-Jae)	
b. Absence of mentors and counselors	All participants lamented the absence of mentors and counselors. Specifically, they never experienced career mentoring and counseling programs while they were competing or in their college years. Although they knew there were some career assistance programs provided by the Ministry of Culture, Sport, and Tourism and some sports organizations, they were not benefited because only high-level athletes such as Olympians, national representatives, and professional athletes could gain opportunities.	General
	Example: It's important to establish academic infrastructure [academic mentoring and counseling in primary and secondary education. And then, the universities should launch academic and professional development programs by fostering professional staff members in their system. Otherwise, there will be ongoing conflicts regarding the current policies and practices. (Dae-Chan)	

General (all or all but one of the cases); typical (more than half the cases); and variant (half the cases or less).

### Factors affecting burning out and terminating athletic careers

Participants in this study shared their own life stories concerning the factors that intentionally and unintentionally caused them to terminate their athletic careers. Everyone had different experiences, and no one single element influenced them to quit their sports; instead, they all faced different challenges that affected their retirement during their college years. Namely, multiplicative factors were intertwined with each other to decrease their motivation to sustain their athletic pursuits, preventing them from continuing their athletic careers. These consisted of injury and failure of rehabilitation (personal inputs and background) as well as bullying and abandonment from coaches and teammates (contextual and environmental issues).

*Injury and Failure of Rehabilitation—*Most participants had various forms of chronic injuries, both serious and minor. In this respect, one of the noticeable factors was the failure of physical rehabilitation. For these reasons, some of the participants who were promising student-athletes burnt out and could not sustain their athletic careers. The case of Guen-Joo, a former basketball student-athlete who was the captain on his team, is an illuminating example of how he could not maintain his athletic career due to injury. The conversation with him through an in-depth interview indicated:

Interviewer: What made you quit your athletic career?

Guen-Joo: I had an ACL [Anterior Cruciate Ligament] problem, so I had to get a knee surgery at the right time, but I couldn't because my coach wouldn't allow me. I endured the pain and injury until my senior year. I left my team, so I couldn't be drafted by a professional team.

Interviewer: So, did you drop out of your athletic program in college?

Guen-Joo: You can say that even if I was registered as an athlete in my senior year.

In-Sung, a former soccer student-athlete who was the captain on his team, was also shared that he got in his senior year and could not be drafted by a professional team. He spoke, “I had multiple ankle injuries before my senior year. I frequently got injured and missed key competitions. I also got injured before I was recruited by the Universiade team which made me quit my sport.” Furthermore, other forms of abusive culture discouraged the participants from sustaining athletic careers. For example, Sang-Jun, a former volleyball player, testified:

Sang-Jun: In my case, I was a national representative at an adolescent level during my high school years, so I thought I had good prospects. However, I suffered from minor injuries in college. However, my coaches or seniors didn't let me take a rest. For these reasons, I burnt out. Also, I had a hard time adjusting to the team, felt a lot of pressure. So, I discussed it with my parents and quit my sport.

Overall, injury and failure to rehabilitate were so unfortunate that most participants in the study encountered various challenges in the athletic culture. They had to endure pain due to the hierarchical social relationships on their teams. For that reason, the participants were frequently challenged to express their basic rights and social needs, accepting a level of unequal treatment from their coaches, seniors, and peers. These factors considerably affected these former college student-athletes.

*Bullying and Abandonment—*Bullying and abandonment are other factors that influenced some of the participants' athletic terminations. In this context, bullying and abandonment mean the participants experienced isolation and exclusion from their coaches and teammates, especially from older/senior athletes, who caused them to leave their teams. In other words, they were excluded rather than being physically punished or verbally abused. Thus, these elements certainly influenced, intentionally or unintentionally, how the participants viewed their athletic terminations even if they were superior athletes. Given this, three participants shared their past experiences.

Byung-Man was a basketball athlete on one of the most high-profile college basketball teams in Korea until his senior year. However, he had limited time to compete as a starting member, which made him feel ignored by coaches and other junior athletes. Hence, he realized that he could not be drafted by professional basketball teams. Byung-Man recalled, “For student-athletes, being drafted by professional sports teams means we are being employed, but I wasn't employed because any professional team recruited me.”.

Furthermore, the story of Dae-Chan demonstrated another example of bullying and abandonment. His case was unique in that he was a professional athlete before being a college athlete. In high school, he was the captain and one of the most extraordinary soccer players at his age level. He explained, “before I went to a professional league, high school athletes usually went to college. However, superior players could go to professional teams instead of college teams.” Dae-Chan was one who competed in the professional league and desired to become a successful professional athlete. When he was on a professional team, the coach who scouted him resigned from his position. For that reason, he was excluded from the roster and eventually released from the team. He further shared, “I eventually joined a [2-years] junior college team and transferred to a 4-years university team. However, I had a difficult time adjusting to the new team because younger athletes were my seniors or in the same cohort…I was often bullied by younger athletes. For these reasons, I left the team before graduation.”.

Moreover, the story of Tae-Soo, who played volleyball, exemplifies a unique example in which he spontaneously quit his sport. Tae-Soo shared:

Tae-Min: I had no scholarship because my coach never provided one for me, so I had to pay for my tuition…My coach never considered me as a regular team member, so I just participated in practice but not in official games. I thought this was sort of meaningless, so I quit volleyball…In college, I wanted to do my best to sustain my athletic career, but I felt like I was sort of excluded by my coach and other teammates. That's why I eventually quit my sport.

Overall, a wide variety of factors perpetuated the participants' sense of burnout that influenced their learning experiences and feelings of exclusion or marginalization from the athletic society altering their outcome expectations. These factors eventually led to their athletic terminations that later led to various forms of life challenges in the career transition process.

### Factors hindering post-retirement career advancement

Participants moved into post-retirement and worked to find new career opportunities. Their career advancement was affected by contextual factors, specifically, prejudice and exclusion, during their career planning and the lack of support from mentors and counselors. These factors affected their self-efficacy and outcome expectations and yet also empowered the participants to help others overcome barriers.

*Prejudice and Exclusion—*Participants were uncertain about their future career planning, and they realized they did not know much about goal setting and had limited academic rigors. Dong-Min said, “I didn't know much about my future career plan and how to start studying after I left my team because I barely attended classes.” Similarly, Chul-Sang shared:

I didn't know much about the class atmosphere and was anxious about being a regular student since I barely came to class… Back in middle and high school, my status was certainly a student, but I think I wasn't a student because I mostly missed class. After all, my athletic and academic schedules overlapped. I didn't even have my school uniforms, which every regular student had to wear. Technically, I would say I wasn't a student while I was a student-athlete.

Due to those diverse obstacles, the participants had lower self-efficacy, which limited their outcome expectations. For instance, Eun-Woo, a basketball athlete, described, “If you say you played sports, many people often think you are not smart enough. Even though you do your best, they overlook your efforts. So, I had to deal with certain prejudice or stereotype made by people. This means, they thought that I don't deserve to be one of their classmates.” Further, Hyo-Chan said, “[un]equal, exclusion? Well, I think so. After I left my team, I had to develop new social and life skills. But it was challenging to adjust to the new society as many people often viewed athletes as an uneducated cohort.” The social context of sport in Korea illustrates the reciprocal relationship between these factors. Due to training and scheduling, athletes have less experience in class, leading to less self-efficacy, which also influenced by the context and specifically by the biased assumption that athletes were ignorant. This, in turn, affected how Korean sport dropout saw their outcome expectations.

Due to social prejudices and exclusions that stigmatize athletes as uneducated and ignorant, they were unable to expand their social networks. Most cited that expanding their social circles outside of athletic culture helped them gain life competencies, collect new information regarding “common knowledge,” and improve social skills for future job searches. Bang-Jin, a soccer player, recognized these issues when he applied to a graduate school and had a job interview:

When I applied to a graduate school, one of the professors said, “I am sorry, I cannot accept you because you were a student-athlete. My class will be progressing in English. How could you catch up to my class?”…It was difficult to get a job. The job recruiters said, “why was your GPA bad?” Next, I did not know how to write a cover letter. I had never learned it. So, I somehow wrote one by myself, but it didn't work.

Tae-Min recalled, “[I]f you confine this into the field of sport, it can be positive. Otherwise, it's not because a lot of people still have prejudice about athletes. Even if athletes have good English skills and good grades, society still has a stereotype that people don't want to hire former athletes. They just think that athletes are not smart enough.” Because of social prejudices and stereotypes about athletes, some of the participants concealed that they were former athletes. Jun-Jae, a former ice hockey player, recalled, “I don't say I was an athlete unless someone asks me.” Chul-Sang, a former soccer player, also recalled, “[T]here were certain prejudices and stereotypes about athletes. People see us as ignorant because we focused solely on athletics and didn't study.” This stigmatization hampered the participants from developing a positive attitude toward their career transitions out of sport. They faced emotional challenges to cultivate social networking circles in their career transition processes. The contextual stigmatization impacted participants and how they had to edit their interests and actions. Due to the outcome expectations, they had to hide a part of their identity.

*Absence of Mentors and Counselors—*When it comes to academic and career barriers and emotional challenges, participants lamented the absence of mentors and counselors who could support their career development. From these experiences, participants developed a want to empower younger college sport dropouts. Min-Jong shared his opinions:

As I did not have my mentors and counselors who could guide me to develop my academic rigor and responsibilities and help me have better opportunities to develop careers, I feel that people who overcame educational, social, or cultural barriers should be mentors for younger sport dropouts. When I meet my juniors, I tell them that no one has the right to say you are ignorant as long as you pursue education to improve your life. I hope I can consult them, but I still don't know how to help them…I believe there should be more appropriate academic mentoring or counseling programs in universities.

Byung-Man also had concerns about younger sport dropouts. He said, “They are too concerned about how to obtain jobs after retirement. They are not confident about their sudden life changes.” He used his experiences to help others, encouraging younger athletes to prepare for their post-retirement careers. He emphasized the importance of providing appropriate academic mentoring or career counseling programs:

Academic mentoring is instrumental. I don't think [sport dropouts] athletes have or develop self-efficacy and had limited time to develop academic rigor and integrity while they were both in academics and athletics. Even though they try their best to do, many of them fall behind and struggle to catch up with their academic tasks. Without career counselors, they cannot easily develop careers.

Overall, participants in the current study perceived that it is significant to encourage younger sport dropouts who often struggle to overcome educational, social, and cultural barriers without academic mentors and counselors. In the absence of support and counselors, these participants chose to help others by demonstrating their resiliency. By reflecting their own experiences, they recognized what others need to overcome barriers and undertook the role of mentors in the career transition processes of other student-athletes.

## Discussion

Although Korea has accomplished a level of success in the global sporting arena by hosting various mega sporting-events (e.g., both the Summer and Winter Olympics, Asian Games, Universiades, FIFA World Cup, and IAAF Championships) and winning numerous medals in those elite events in the past four decades, cultural emphasis on winning is strong, as the coaching and athlete relationships are closely entwined in power dynamics (Kim et al., 2020; Kim and Dawson, 2021; Kim and Tak, 2021). The power hierarchical relationships entail various issues in the athletic system, pertaining to: (1) physical violence and punishment; (2) sexual harassment and gender discrimination against female athletes; (3) deprivation of academic freedom; and (4) biased judgments, match-fixing, and bribery concerning high school athletes' college admissions at large (Park et al., 2012; Lim et al., 2015).

The issues of educational rights directly influence career transition issues among Korean athletes and hinder them from gaining competitive occupational opportunities. Notably, the high dropout rate of elite college student-athletes and their limited opportunities to obtain white-collar and full-time employment opportunities are growing concerns for the national sport movement (KUSF, 2017, 2018, 2019). In this context, participants in the current study outlined their experiences with injury, abandonment, bullying, and prejudices as they transitioned from student-athlete to student to worker. These experiences had reciprocal interactions with athletes' predispositions, background, and context and influenced their learning experiences and outcome expectations throughout their career development.

The participating student-athletes used their experiences to develop support for other student-athletes and sport dropouts, showing not only their resiliency but also their ability to empower others. SCCT aids in understanding how to create goals, overcome barriers, and embrace empowerment; participants were able to empower others to move through oppressive systems which devalued their work as athletes and create fewer financial opportunities, by sharing both strengths and barriers with others. The participating student-athletes also highlighted how they used their experiences to develop support for other student-athletes and sport dropouts, showing not only their resiliency to survive but also their ability to empower others to survive.

### Theoretical interpretations: Using SCCT to determine the burnout and dropout factors

Participants who experienced burnout and athletic career termination also experienced a lack of support during their injury rehabilitation. Health status, such as injury, is a personal input that influenced the learning expectations. Participants did not receive support when injured, and their worth was tied to only to their athletic performance. Hence, their athletic self-efficacy decreased, and their outcome expectations no longer supported athletics as a career. Podlog et al. (2011) also found that sports injury impairs athletes' sense of competence, autonomy, and social interaction. Participants in this study perceived the lack of self-efficacy and social support as they found themselves financially unsupported, abandoned, and ignored by their coaches and team. The lack of social support highlights an area of intervention for counselors. Therefore, in developing support for injured student-athletes, it is significant to consider their fundamental needs and the ways to protect their identity as an athlete and student as well as strengthen social and emotional support from their teams and coaches. Yang et al. (2010) highlighted an urgent need to define the psychosocial needs of injured athletes better and realize that athletic trainers, coaches, and teams are essential in exploring that work.

SCCT outlines psychosocial support as building opportunities for self-efficacy and outcome expectations through collaboration and context (Chronister and McWhirter, 2003). These specific factors can help our student-athletes feel empowered in their career transitions and support their overall wellbeing. Student-athletes need to develop an identity as both an athlete and a student early in their careers, calling for collaboration across athletics and academics. Teachers, coaches, athletic trainers, fellow athletes, family, and counselors can work together to build a support network (Yang et al., 2010) that would address academic, financial, social, psychological, and physical concerns and would equip athletes with the resources they need as they heal from injury and deal with financial concerns (Podlog et al., 2011).

Overall, the support network also instills a sense of community and keeps players connected to the sport and team. With added social support and resources, student-athletes can learn that their worth is not solely dependent on what they can produce in the athletic arena, but how they support each other and engage in the network. This reciprocal relationship builds a context of support rather than abandonment, regardless of career choices. It is important to support student-athletes as they make career decisions regarding their future in both sports and academics. The goal is to cultivate a culture in which sport and academics are no longer at odds with each other and promote the same goals for their students.

Additionally, as participants moved into post-retirement and career attainment and search, they found prejudice and exclusionary practices in both academic and career settings. They felt that their skills as students and workers with their identity as athletes were not socially accepted, prompting some to abandon the identity of an athlete to succeed in the job market. The college sport dropouts in this study were able to gain careers and break stereotypes about their abilities only by hiding their athletic identity, demonstrating the system of oppression that surrounds their career transition. Therefore, to support self-efficacy, it is crucial to contemplate the need to use both context and competency to improve interventions (Chronister and McWhirter, 2003). In reflecting on their past, college sport dropouts were able to outline the various aspects needed to succeed in career transition and make recommendations for others to succeed by tackling both surrounding prejudices (context) and recognizing skills and resources (competency).

### Practical implications: Using SCCT to empower college sport dropouts

As aforementioned, participants highlighted the lack of support during their career transition. Once they are off the athletic team and out of school, they become stuck without the support and with unclear identity or career pathway. Counselors should explore transitional programs to support the career development of student-athletes during this time. These programs should create a motivational climate that would support exploration and meet these students' contextual, social, and psychological needs (Calvo et al., 2010). Specifically, these needs can be addressed by helping sport dropouts gain competency through recognizing transferable skills, developing new skills, and engaging with resources that will aid in their career attainment (Chronister and McWhirter, 2003). Given the context and social prejudices that student-athletes face, their current skills could be used to form new skills to combat psychological symptoms, such as negative self-image, isolation, and identity crisis, which plague sport dropouts during their transition (Cosh et al., 2013; Crane and Temple, 2015; Roberts et al., 2015). Counselors can work to improve self-efficacy, create specific programs to address these issues, and work on preventive measures.

Participants in this study were also active in helping and mentoring other student-athletes and sport dropouts by modeling their successes and outlining their experiences with overcoming barriers. Therefore, counselors can extend the support network to include mentors who both empower student-athletes and sport dropouts and break stereotypes by modeling success, creating room for empowerment and resiliency. Galli and Vealey (2008), found that there are general dimensions describing athletes' resilience experiences including: sociocultural influences, personal resources, and positive outcomes. Therefore, those working with student athletes and can support resiliency through programs designed through SCCT, which focuses on social, cultural, personal, and outcome expectations. Building a support network, connecting to mentors, and organizing programs for sport dropouts help (1) identify accomplishments and skills; (2) engage students in vicarious learning; (3) increase exposure to positive feedback; and (4) provide strategies for managing psychological symptoms. All these interventions aid students to survive in a system of oppression. However, due to the reciprocal relationship, there is hope that these interventions will promote social and cultural changes.

### Limitations and future research

This study had a few limitations. First, the sample only heard about men's experiences of sport. Future research should explore career transition experiences of other genders. Second, participants in this study were only playing team sports; thus, this study did not investigate the experiences of athletes in individual competitive sports. Future research can explore support structures offered to those engaged in individual competitive sports. This study was exploratory and used SCCT to guide helpful interventions. Future research should explore SCCT in a quantitative study to investigate self-efficacy within the context of Korean sport dropout. With this generalizable information, both intervention and policy can be developed and implemented. Additionally, a longitudinal study could highlight more ways in which student-athletes can be supported in their career transitions. Unfortunately, this study could not include former female student-athletes. The current KUSF system which is the official college sport authority oversees male sports such as soccer, basketball, baseball, volleyball, and ice hockey. The authors, of course acknowledge that there are more individual sports, but primarily included male team sports to draw coherent findings. Therefore, future researchers can consider including female team sports as well as individual sports regardless of gender. Overall, more research needs to be done to explore specific policy and advocacy efforts to aid in these cultural transformations.

## Concluding remarks

This study explored the experiences of college student dropouts with oppression and their resilience as well as outlined opportunities for empowerment. The findings identified an urgent need to better support student-athletes and sport dropouts. This study used SCCT to understand their experiences and guide specific interventions that can be put into action for current student-athletes and those transitioning from a student-athlete identity. School and career counselors can work with multiple systems to support self-efficacy and outcome expectations. This study amplifies the voices of college sport dropouts who are surviving in systems that oppress their educational rights and do not provide infrastructural support for career development. The current study makes suggestions on how counselors can work within these systems to aid career transitions. However, this study also calls upon researchers, counselors, and administrators to continue exploring advocacy efforts with this population to alter policy and practice. This study highlighted the various social issues ingrained in the athletic system's social hierarchy, which contributes to oppressive systems. This study emphasizes the strengths of those who survived these oppressive systems and outlines ways to use SCCT to increase resiliency and empowerment. Therefore, the current study hopes to inform future efforts to not only help sport dropouts survive but also transform oppressive systems into supportive ones.

## Data availability statement

The datasets presented in this article are not readily available because data can be provided upon reasonable request and is constrained by anonymity issues. Requests to access the datasets should be directed to BN, W2004@shisu.edu.cn.

## Ethics statement

The studies involving human participants were reviewed and approved by Office of Research at the University of Tennessee, Knoxville. The patients/participants provided their written informed consent to participate in this study.

## Author contributions

BN was in charge of designing this study, collecting data, and analyzing the data. RM was in charge of adopting the theoretical framework and interpreting the findings. All authors contributed to the article and approved the submitted version.

## Conflict of interest

The authors declare that the research was conducted in the absence of any commercial or financial relationships that could be construed as a potential conflict of interest.

## Publisher's note

All claims expressed in this article are solely those of the authors and do not necessarily represent those of their affiliated organizations, or those of the publisher, the editors and the reviewers. Any product that may be evaluated in this article, or claim that may be made by its manufacturer, is not guaranteed or endorsed by the publisher.

## Footnotes

^1^Purposive and snowball sampling methods are useful for researchers who have difficulties recruiting analogous samples, allowing participants to introduce or recruit similar or best-matched participants (Merriam and Tisdell, 2016). The initial four participants were recruited through personnel from the KUSF. The initially recruited participants introduced their friends or alumni to the primary author.
